# Accuracy of different diagnostic tests for early, delayed and late prosthetic joint infection

**DOI:** 10.1186/s12879-017-2693-1

**Published:** 2017-08-25

**Authors:** M. Fernández-Sampedro, C. Fariñas-Alvarez, C. Garces-Zarzalejo, M. A. Alonso-Aguirre, C. Salas-Venero, L. Martínez-Martínez, M. C. Fariñas

**Affiliations:** 1 Infectious Diseases Unit, Department of Medicine, Hospital Universitario Marqués de Valdecilla-IDIVAL, School of Medicine, University of Cantabria, Santander, Spain; 2 Division of Health Care Quality Hospital, Hospital Universitario Marqués de Valdecilla-IDIVAL, School of Medicine, University of Cantabria, Santander, Spain; 3 Department of Orthopaedic Surgery, Hospital Universitario Marqués de Valdecilla-IDIVAL, School of Medicine, University of Cantabria, Santander, Spain; 4 Service of Microbiology, Hospital Universitario Marqués de Valdecilla-IDIVAL, School of Medicine, University of Cantabria, Santander, Spain

## Abstract

**Background:**

A combination of laboratory, histopathological and microbiological tests for diagnosis of prosthetic joint infection (PJI) have been strongly recommended. This study aims to characterize the accuracy of individual or group tests, such as culture of sonicate fluid, synovial fluid and peri-implant tissue, C-reactive protein (CRP) and histopathology for detection of early, delayed and late PJI.

**Methods:**

A prospective study of patients undergoing hip or knee arthroplasty from February 2009 to February 2014 was performed in a Spanish tertiary health care hospital. The diagnostic accuracy of the different methods was evaluated constructing receiver-operating-characteristic (ROC) curve areas.

**Results:**

One hundred thirty consecutive patients were included: 18 (13.8%) early PJI, 35 (27%) delayed PJI and 77 (59.2%) late PJI. For individual parameters, the area under the ROC curve for peri-implant tissue culture was larger for early (0.917) than for delayed (0.829) and late PJI (0.778), *p* = 0.033. There was a significantly larger difference for ROC area in the synovial fluid culture for delayed (0.803) than for early (0.781) and late infections (0.679), *p* = 0.039. The comparison of the areas under the ROC curves for the two microbiological tests showed that sonicate fluid was significantly different from peri-implant tissue in delayed (0.951 vs 0.829, *p* = 0.005) and late PJI (0.901 vs 0.778, *p* = 0.000)*.* The conjunction of preoperative parameters, synovial fluid culture and CRP, improved the accuracy for late PJI (*p* = 0.01). The conjunction of histopathology and sonicate fluid culture increased the area under ROC curve of sonication in early (0.917 vs 1.000); *p* = 0.06 and late cases (0.901 vs 0.999); *p* < 0.001.

**Conclusion:**

For early PJI, sonicate fluid and peri-implant tissue cultures achieve the same best sensitivity. For delayed and late PJI, sonicate fluid culture is the most sensitive individual diagnostic method. By combining histopathology and peri-implant tissue, all early, 97% of delayed and 94.8% of late cases are diagnosed. The conjunction of histopathology and sonicate fluid culture yields a sensitivity of 100% for all types of infection.

## Background

The successful management of patients with prosthetic joint infection (PJI) depends on an early and accurate diagnosis. Despite significant progress in the diagnosis of PJI, there is currently no single routinely used clinical or laboratory test that achieves optimal diagnostic accuracy [1]. Over the last several years great advances have been made and several workgroups have proposed definitions for PJI, however, none have been widely adopted [2–5]. Guideline limitations in providing evidence are highlighted by the heterogeneity of the definitions and classifications, and at times, conflicting criteria have been proposed [2–5]. In order to optimize the diagnosis of implant-associated infection, a combination of clinical findings, laboratory results from peripheral blood and synovial fluid, microbiological data, histological evaluation of periprosthetic tissue, intraoperative inspection, and, in some cases radiographic results is considered [6]. However, test performance may vary with joint type and with post-arthroplasty implantation removal time. There have been several studies reported in the literature dealing with the evaluation of sensitivity and specificity of different diagnostic methods for implant-related infections, however, there is a lack of published data outlining their usefulness based on the time to infection [7, 8].

The aim of this study was to compare the diagnostic accuracy of the individual or grouped tests such as sonicate fluid, synovial fluid and peri-implant tissue culture, C reactive protein (CRP) and histopathology for the detection of early, delayed and late PJI, as this would allow us to enhance the diagnosis of each type of orthopedic implant infection.

## Methods

The study was conducted in a tertiary health care hospital, which performs approximately 500 hip and 300 knee arthroplasties per year. From February 2009 to February 2014, all consecutive patients aged 18 years or older who underwent a total or partial hip or knee arthroplasty revision at our institution were prospectively logged and followed-up in a database.

Patients were excluded from the study if fewer than two peri-implant tissue samples were submitted for culture, if the implant was not obtained or if an evident contamination of the implant was identified in the operating room. Patients who underwent chemotherapy in the 60 days prior to index surgery and with recent periprosthetic fractures were also excluded, due to the acute stress of the treatment that might elevate the neutrophil count number in the histopathology [9].

PJI was diagnosed according to the criteria proposed by Berbari et al. by the presence of at least one of the following [10]: (i) visible purulence surrounding the prosthesis, (ii) acute inflammation on histopathologic examination of permanent tissue sections, (iii) a sinus tract communicating with the prosthesis (iv) two or more cultures of joint aspirates or cultures of intraoperative tissue specimens yielded the same microorganism when *S. aureus* or *S. lugdunensis* were the microorganisms isolated, only a single positive tissue specimen was required.

Our study did not included sonicate fluid culture (SFC) as diagnosis criteria for infection, however, we evaluate the detection validity of SFC for PJI to provide further evidence for its clinical use. The histopathological criteria established by Morawietz and Krem was used to define a standardized evaluation of the periprosthetic membrane [11]. Since erythrocyte sedimentation rate (ESR), CRP and white blood cell count are part of our clinical routine; a preoperative determination was performed 2 weeks before the surgery. According to the onset of symptoms after implantation, a postoperative infection was considered “early” when PJI occurred less than 3 months after implantation, “delayed” when PJI occurred between 3 and 12 months and “late” when PJI occurred >12 months after the prosthesis implantation [2, 12].

Demographics, co-morbid conditions, type of implant, surgical procedure, antimicrobial treatment and outcome were prospectively gathered using a standardized data collection form. Previous antimicrobial therapy was defined as the administration of antibiotic treatment within the 14-day period before the removal of the orthopaedic implant.

### Specimen collection, cultures and sonication

Three to 6 deep samples of periprosthetic tissue with the most evident inflammatory changes were collected for histopathology and conventional microbiologic culture. Removed orthopedic implants were sonicated as previously described, and a cutoff of ≥20 colony-forming unit (CFU) per plate was applied to determine a clinically significant result as described previously [13–15]. In addition, a set of blood cultures was collected if the body temperature was higher than 38 °C or when shaking chills were present.

### Statistical analysis

Statistical analysis was performed using a two-tailed χ^2^ test and a Fisher’s exact test, or ANOVA test, as appropriate in each case. For m × n tables, a Fisher’s exact test was estimated using the Monte Carlo method. Sensitivity, specificity and positive and negative predictive values were calculated. Ninety-five percent confidence intervals (95% CI) were calculated as exact binomial confidence intervals. Based on receiver-operating-characteristic (ROC) analysis of the data, the cut-off level for CRP was >1.

The combination of different diagnostic tests was performed using an “or” combination. The combined test acted as a new test: in scheme “or”, two of two negative tests were necessary to consider the composite diagnostic as negative. To outline the composite diagnostic the combination schemes for the diagnostic tests were as follows: i) test 1 positive and test 2 positive, composite diagnostic positive; ii) test 1 positive and test 2 negative, composite diagnostic positive; iii) test 1 negative and test 2 positive, composite diagnostic positive; iv) test 1 negative and test negative, composite diagnostic negative [16]. Under this scheme, sensitivity increases while specificity decreases.

The diagnostic accuracy of the different methods was evaluated by constructing ROC curve areas computed using the trapezoidal rule. De Long et al. [17], method was used for comparison between ROC areas.

A *p*-value of less than 0.05 (two-sided) was considered statistically significant. Calculations were performed with the SPSS package v19.0 (SPSS Inc., Chicago, Illinois), and the Stata statistical software (Release 11.0, Stata Corporation, College Station, TX).

## Results

A total of 504 patients were prospectively included during the study-period. Six patients (1.2%) were excluded: 3 because no implant was submitted to microbiology laboratory, 2 because of a recent periprosthetic fracture, and 1 who underwent chemotherapy treatment for breast cancer. Of the remaining 498 patients, 130 (26.10%) were finally diagnosed with infection and, among these, 18 (13.8%) were classified as early, 35 (27%) as delayed and 77 (59.2%) as late PJI. The demographic characteristics of the patients are shown in Table 1.

**Table 1:** Characteristics of patients with prosthetic joint infection

	Early (< 3 months) *n* = 18	Delayed (3–12 months) *n* = 35	Late (>12 months) *n* = 77	*p*-value*
Demographics				
Mean age (yrs) (IQR)**	65.05 (42–83)	62.2 (31–84)	67.2 (33–88)	0.088
Gender Female-no (%)	8 (44.4%)	20 (57.1%)	36 (46.7%)	0.540
Type of implant				
Knee -no (%)	3 (16.7%)	14 (40%)	29 (37.7%)	
Hip-no (%)	15 (83.3%)	21 (60%)	48 (62.3%)	0.196
Antibiotic prior to revision surgery-no (%)	10 (55%)	6 (17.1%)	9 (11.6%)	<0.0001
Type of surgery				0.081
Two stage revision	6 (33.3%)	21 (60%)	43 (55.8%)	
One stage revision	1 (5.5%)	5 (14.3%)	9 (11.7%)	
Partial revision	11 (61.2%)	7 (20%)	24 (31.2%)	
Girdlestone	0	2 (5.7%)	1 (1.3%)	

*yrs* years

*Two-tailed ANOVA test, χ^2^ test or Fisher exact test as corresponding

**IQR interquartile range

There were two aseptic failure cases with positive sonicate fluid cultures. None of them did receive antibiotics beyond standard peri-operative prophylaxis. One case, with prosthesis failure between 3 and 12 months after the last implant placement, had isolation of >100 CFU of coagulase-negative staphylococci (CNS) and, after 6 years of follow up, he has a functional recovery and independent walking. The other case, which failed over 12 months after the last implantation, had *Enterococcus faecalis* isolation and the patient underwent repeat revision arthroplasty due to suspected implant-related infection 16 months later. The results of microbiological and histopathological examinations were negative. At the present time, she needs to walk on two crutches due to aseptic loosening in the opposite symptomatic knee arthroplasty but she does not wish to undergo surgery. According to the definition of PJI, these two cases were classified as false positives and were not included in the 130 PJI defined cases.

### Comparison of individual diagnostic parameters for the diagnosis of PJI

Table 2 shows the results comparing the culture of sonicate fluid, synovial fluid and peri-implant tissue, the CRP and the histopathology of early, delayed and late infections in terms of accuracy measures.

**Table 2:** Comparison of sensitivity, specificity, PPV, and NPV for individual parameters in 498 patients with aseptic loosening and prosthetic joint infection (18 early, 35 delayed and 77 late)

Diagnostic test	AL	PJI	Specificity (%)	Sensitivity (%)	PPV (%)	NPV (%)	ROC área (95% CI)	*P*-value*
	TN/total	TP/total	(95% CI)	(95% CI)	(95% CI)	(95% CI)		
CRP (>1)								
All patients	310/367	90/128	84.5 (80.4–88.0)	70.3 (61.6–78.1)	61.2 (52.8–69.1)	89.1 (85.3–92.2)	0.774 (0.730–0.818)	0.124
Early	10/13	13/16	76.9 (46.2–95.0)	81.3 (54.4–96.0)	81.3 (54.4–96.0)	76.9 (46.2–95.0)	0.791 (0.636–0.946)	
Delayed	20/23	29/35	87.0 (66.4–97.2)	82.9 (66.4–93.4)	90.6 (75.0–98.0)	76.9 (56.4–91.0)	0.849 (0.754–0.943)	
Late	280/331	48/77	84.6 (80.2–88.3)	62.3 (50.6–73.1)	48.5 (38.3–58.7)	90.6 (86.8–93.6)	0.735 (0.678–0.792)	
Synovial fluid culture								
All patients	310/310	53/116	100 (98.8–100)	45.7 (36.4–55.2)	100 (93.3–100)	83.1 (78.9–86.8)	0.728 (0.683–0.774)	0.039
Early	13/13	9/16	100 (75.3–100)	56.3 (29.9–80.2)	100 (66.4–100)	65.0 (40.8–84.6)	0.781 (0.656–0.906)	
Delayed	18/18	20/33	100 (81.5–100)	60.6 (42.1–77.1)	100 (83.2–100)	58.1 (39.1–75.5)	0.803 (0.718–0.888)	
Late	279/279	24/67	100 (98.7–100)	35.8 (24.5–48.5)	100 (85.8–100)	86.6 (82.4–90.2)	0.679 (0.621–0.736)	
Peri-implant tissue culture								
All patients	367/368	81/130	99.7 (98.5–100)	62.3 (53.4–70.7)	98.8 (93.4–100)	88.2 (84.7–91.2)	0.810 (0.768–0.852)	0.033
Early	13/13	15/18	100 (75.3–100)	83.3 (58.6–96.4)	100 (78.2–100)	81.3 (54.4–96.0)	0.917 (0.828–1.000)	
Delayed	24/24	23/35	100 (85.8–100)	65.7 (47.8–80.9)	100 (85.2–100)	66.7 (49.0–81.4)	0.829 (0.749–0.908)	
Late	330/331	43/77	99.7 (98.3–100)	55.8 (44.1–67.2)	97.7 (88.0–99.9)	90.7 (87.2–93.4)	0.778 (0.722–0.834)	
Sonicate fluid culture								
All patients	366/368	110/130	99.5 (98.1–99.9)	84.6 (77.2–90.3)	98.2 (93.7–99.8)	94.8 (92.1–96.8)	0.920 (0.889–0.952)	0.402
Early	13/13	15/18	100 (75.3–100)	83.3 (58.6–96.4)	100 (78.2–100)	81.3 (54.4–96.0)	0.917 (0.828–1.000)	
Delayed	23/24	33/35	95.8 (78.8–99.9)	94.3 (80.8–99.3)	97.1 (84.7–99.9)	92.0 (74.0–99.0)	0.951 (0.894–1.000)	
Late	330/331	62/77	99.7 (98.3–100)	80.5 (69.9–88.7)	98.4 (91.5–100)	95.7 (92.9–97.5)	0.901 (0.856–0.946)	
Histology								
All patients	368/368	100/128	100 (99.0–100)	78.1 (70.0–84.9)	100 (96.4–100)	92.9 (89.9–95.3)	0.891 (0.855–0.927)	0.190
Early	13/13	14/18	100 (75.0–100)	77.8 (52.4–93.6)	100 (76.8–100)	76.5 (50.1–93.2)	0.889 (0.790–0.987)	
Delayed	24/24	29/33	100 (85.8–100)	87.9 (71.8–96.6)	100 (88.1–100)	85.7 (67.3–95.9)	0.939 (0.883–0.996)	
Late	331/337	57/77	100 (98.9–100)	74.0 (62.8–83.4)	100 (93.7–100)	94.3 (91.3–96.4)	0.870 (0.821–0.919)	

*AL* aseptic loosening, *PJI* prosthetic joint infection, *TN* true negative, *TP* true positive, *CI* confidence interval, *PPV* positive predictive value, *NPV* negative predictive value, *CRP* C-reactive protein

*Two sided *P*-value Test of equality of independent ROC areas in the three groups of patients with PJI: early, delayed and late

According to individual parameters, in early PJI, both peri-implant tissue and sonicate fluid culture reached the same best sensitivity of 83.3% followed by CRP and the histology with 81.3% and 77.8%, respectively. For delayed PJI, the highest sensitivity was obtained with sonicate fluid culture with 94.3% followed by histology, CRP and peri-implant tissue culture with 87.9%, 82.9% and, 65.7%, respectively. In the case of late PJI, the highest sensitivity was found for the sonicate fluid culture with 80.5% followed by histology, CRP, and peri-implant tissue culture with 74%, 62.3% and 55.8%, respectively.

The area under the ROC curve for peri-implant tissue culture was larger for early infections than for delayed and late PJI: 0.917 vs. 0.829 vs. 0.778 respectively, *p* = 0.033. There was also a significant difference for ROC area in the synovial fluid culture for delayed PJI compare to early and late infections: 0.803 vs. 0.781 vs. 0.679 respectively, *p* = 0.039.

The comparison of the areas under the ROC curves for the two microbiological tests showed that sonicate fluid culture was significantly different from peri-implant tissue culture in delayed (0.951 vs 0.829, *p* = 0.005) and late PJI (0.901 vs 0.778, *p* = 0.000).

When comparing ROC curves for the two preoperative parameters, CRP and synovial fluid culture, there was no significant difference in accuracy for any type of PJI, nor were any significant differences found when comparing histology and sonicate fluid culture.

### Comparison of grouped diagnostic parameters for PJI diagnosis

To further improve the diagnostic validity combinations two tests were performed. Table 3, shows the comparison of accuracy measures for sensitivity, specificity, positive predictive value (PPV), negative predictive value (NPV), and the ROC curve for early, delayed and late PJI.

**Table 3:** Diagnostic Accuracy for Grouped Parameters in 498 Patients with Aseptic Loosening and Prosthetic Joint Infection (18 early, 35 delayed and 77 late)

	AL	PJI	Specificity (%)	Sensitivity (%)	PPV (%)	NPV (%)	ROC area	*P*-value*
	TN/total	TP/total	(95% CI)	(95% CI)	(95% CI)	(95% CI)	(95% CI)	
CRP + Synovial fluid culture								
All patients	261/309	91/114	84.5 (79.9–88.3)	79.8 (71.3–86.8)	65.5 (56.9–73.3)	91.9 (88.1–94.8)	0.822 (0.779–0.863)	0.073
Early	10/13	12/14	76.9 (46.2–95.0)	85.7 (57.2–98.2)	80.0 (51.9–95.7)	83.3 (51.6–97.9)	0.813 (0.660–0.966)	
Delayed	14/17	30/33	82.4 (56.6–96.2)	90.9 (75.7–98.1)	90.9 (75.7–98.1)	82.4 (56.6–96.2)	0.866 (0.760–0.972)	
Late	237/279	49/67	84.9 (80.2–88.9)	73.1 (60.9–83.2)	53.8 (43.1–64.4)	92.9 (89.1–95.8)	0.790 (0.733–0.848)	
CRP + Peri-implant tissue culture								
All patients	310/367	110/128	84.5 (80.4–88.0)	85.9 (78.7–91.4)	65.9 (58.1–73.0)	94.5 (91.5–96.7)	0.852 (0.817–0.888)	0.331
Early	10/13	16/16	76.9 (46.2–95.0)	100 (79.4–100)	84.2 (60.4–96.6)	100 (69.2–100)	0.885 (0.765–1.000)	
Delayed	20/23	32/35	87.0 (66.4–97.2)	91.4 (76.9–98.2)	91.4 (76.9–98.2)	87.0 (66.4–97.2)	0.892 (0.807–0.977)	
Late	280/331	62/77	84.6 (80.2–88.3)	80.5 (69.9–88.7)	54.9 (45.2–64.2)	94.9 (91.8–97.1)	0.826 (0.777–0.874)	
CRP + Sonicate fluid culture								
All patients	309/367	119/128	84.2 (80.1–87.8)	93.0 (87.1–96.7)	67.2 (59.8–74.1)	97.2 (94.7–98.7)	0.886 (0.857–0.915)	0.532
Early	10/13	15/16	76.9 (46.2–95.0)	93.8 (69.8–99.8)	83.3 (58.6–96.4)	90.9 (58.7–99.8)	0.853 (0.719–0.987)	
Delayed	20/23	34/35	87.0 (66.4–97.2)	97.1 (85.1–99.9)	91.9 (78.1–98.3)	95.2 (76.2–99.9)	0.921 (0.845–0.996)	
Late	279/331	70/77	84.3 (79.9–88.0)	90.9 (82.2–96.3)	57.4 (48.1–66.3)	97.6 (95.0–99.0)	0.876 (0.838–0.914)	
Histology + Peri-implant tissue culture								
All patients	367/368	123/128	99.7 (98.5–100)	96.1 (91.1–98.7)	99.2 (95.6–100)	98.7 (96.9–99.6)	0.979 (0.962–0.996)	0.061
Early	13/13	18/18	100 (75.3–100)	100 (81.5–100)	100 (81.5–100)	100 (75.3–100)	1.000 (1.000–1.000)	
Delayed	24/24	32/33	100 (85.8–100)	97.0 (84.2–99.9)	100 (89.1–100)	96.0 (79.6–99.9)	0.985 (0.955–1.000)	
Late	330/331	73/77	99.7 (98.3–100)	94.8 (87.2–98.6)	98.6 (92.7–100)	98.8 (97.0–99.7)	0.973 (0.947–0.998)	
Histology + Sonicate fluid culture								
All patients	366/368	128/128	99.5 (98.1–99.9)	100 (97.2–100)	98.5 (94.3–99.8)	100 (99.0–100)	0.997 (0.993–1.000)	0.37
Early	13/13	18/18	100 (75.3–100)	100 (81.5–100)	100 (81.5–100)	100 (75.3–100)	1.000 (1.000–1.000)	
Delayed	23/24	33/34	95.8 (78.9–99.9)	100 (89.4–100)	97.1 (84.7–99.9)	100 (85.2–100)	0.978 (0.938–1.000)	
Late	330/331	77/77	99.7 (98.3–100)	100 (95.3–100)	98.7 (93.1–100)	100 (98.9–100)	0.999 (0.996–1.000)	
Peri-implant tissue culture + Sonicate fluid culture								
All patients	365/368	111/130	99.2 (97.6–99.8)	85.4 (78.1–91.0)	97.4 (92.5–99.5)	95.1 (92.4–97.0)	0.923 (0.892–0.954)	0.32
Early	13/13	16/18	100 (75.3–100)	88.9 (65.3–98.6)	100 (79.4–100)	86.7 (59.5–98.4)	0.945 (0.870–1.000)	
Delayed	23/24	33/34	95.8 (78.9–99.9)	94.3 (80.8–99.3)	97.1 (84.7–99.9)	92.0 (74.0–99.0)	0.951 (0.894–1.000)	
Late	329/331	62/77	99.4 (97.8–99.9)	80.5 (69.9–88.7)	96.9 (96.2–99.6)	95.6 (92.9–97.5)	0.900 (0.855–0.944)	

*AL* aseptic loosening, *PJI* prosthetic joint infection, *TN* true negative, *TP* true positive, *CI* confidence interval, *PPV* positive predictive value, *NPV* negative predictive value, *CRP* C-reactive protein

*Two sided *P*-value Test of equality of independent ROC areas in the three groups of patients with PJI: early, delayed and late

The conjunction of preoperative parameters such as synovial fluid culture and CRP significantly improved the accuracy of individual diagnosis of CRP in detection of late PJI 0.735 vs. 0.790, (*p* = 0.01) but not that of early PJI (*p* = 0.64), however, delayed PJI had a near-significant trend, 0.849 vs. 0.866, (*p* = 0.07).

The addition of CRP to peri-implant tissue culture improved accuracy of individual diagnosis of CRP for all the types of PJI this being especially significant in late PJI [early 0.791 to 0.852, (*p* = 0.06); delayed 0.849 to 0.892, (*p* = 0.07); and late 0.735 to 0.826, (*p* < 0.0001)]. On the other hand, the conjunction of CRP and sonicate fluid culture did not improve the individual diagnosis of sonicate fluid for any type of PJI.

The conjunction of histopathology and sonicate fluid culture yielded a sensitivity of 100% for early, delayed and late cases. This combination allowed the area under ROC curve of sonicate fluid culture to increase in early cases from 0.917 to 1.000 (*p* = 0.06), in delayed cases from 0.951 to 0.978 (*p* = 0.15) and in late cases from 0.901 to 0.999 (*p* < 0.001).

By combining histopathology and peri-implant tissue culture, 100% of early, 97% of delayed and 94.8% of late cases were diagnosed. This combination allowed the area under ROC curve of peri-implant tissue culture achieved in early cases to increase from 0.917 to 1.000 (*p* = 0.06), in delayed from 0.829 to 0.985 (*p* < 0.001) and in late cases from 0.778 to 0.973 (*p* < 0.001).

The addition of peri-implant tissue to sonicate fluid culture did not improve the individual diagnostic performance of sonicate fluid culture. The areas under the two ROC curves were the same in delayed and late PJI, and were very similar in early PJI.

### Microbiology

One hundred and eleven of the 130 patients diagnosed with PJI showed a positive bacterial culture.

For early infections, CNS and gram negative bacilli (GNB) were the main isolated microorganisms (47.6% and 38%, respectively), and *S. aureus* (4.7%) was the least prevalent bacteria. In the case of delayed infections, CNS was the most frequently found microorganism (52%) followed by GNB (20%) and *S. aureus* and *Streptococcus* spp. (12% each of them). In late infections, CNS was cultured in 70.5% of cases followed by *S. aureus* (6.6%) and GNB (4.4%).

Regarding infections due to multi-resistant bacteria, there were only 2 isolations with methicillin resistant *S. aureus* in the delayed group and 1 with *E. coli* producing an extended-spectrum beta-lactamase (ESBL) in early PJI.

For delayed PJI, in the sonicate fluid culture SCN was isolated in 19 patients and Streptococci in 5 patients, whereas in the peri-implant tissue culture SCN was isolated in only 13 patients and Streptococci in 3.

For late PJI, in the sonicate fluid culture SCN was isolated en 43 patients and Streptococci in 3 patients, whereas in the peri-implant tissue culture SCN was isolated in only 35 patients and there was no isolation of Streptococci.

## Discussion

The present study assesses the accuracy of the individual or grouped diagnostic tests for PJI, including CRP, histopathology and microbiology tests, based on the time of infection after prosthesis implantation. So far, this is the first study, which compares diagnosis tests according to onset of PJI taking into account Osmon criteria [2].

Regarding individual methods, our study found that both peri-implant tissue and sonicate fluid cultures are highly sensitive and reach the same accuracy for diagnosis of early PJI. This finding might be related to the microorganisms isolated, as CNS, a slow growing and biofilm-producing microorganism caused nearly half of the early cases (47.6%), which may explain the high sensitivity of implant sonication. For delayed and late infections, sonicate fluid culture was the most sensitive diagnostic method studied. Consistent with our findings, a study comparing peri-implant tissue and sonicate fluid cultures found significantly higher sensitivity of implant sonication in cases of chronic infections but not in acute infections. Notwithstanding, in that study delayed and late infections were grouped together since treatment and prognosis are similar [18].

Actually, with the introduction of new diagnostic methods, there have been many cases and much uncertainty about the interpretation of positive cultures with these new techniques. Some authors reported a higher sensitivity from standard cultures than from sonicate fluid cultures [19] while others agree that sonication increases the diagnostic accuracy for implant-related infection [13, 20]. Several studies observed that antibiotic intake prior to prosthetic removal negatively affected the microbial detection of tissue and sonication cultures [20–22], while others had no influence [18, 23, 24]. We found differences in bacteria detection when antibiotics had been previously used for all types of infection. It was also found that more than one-half (55.5%) of early PJI patients had received antibiotic therapy within 14 days before the surgery date according to the discretion of the treating physician.

In addition, the clinical implications, together with the different results between studies, show that cultures performed in the clinical microbiology laboratory vary between centers depending in a variety of factors including specimen processing period, prosthetic solution, geographical location, anaerobic culture period and the use of centrifugation and vortexing. A recent meta-analysis of sonication fluid samples showed that 14-day anaerobic culture may improve sensitivity, the use of centrifugation or vortexing may improve specificity and the use of 400 to 500 ml of Ringer’s solution for containers may improve sensitivity and specificity [25]. Therefore, it may be useful to evaluate the sensitivity of the clinical microbiology laboratory before implementing a new technology.

Our results identified histopathology as showing the second highest sensitivity out of all the individual parameters in delayed and late infections. Some studies have been able to demonstrate the superiority of the histological diagnosis of PJI over microbiological methods, however, ultrasonication was not used as an add-on test for routine microbiological diagnostic [26–28]. In contrast, Janz et al. [29] found that sonication was able to improve the sensitivity of the histological analysis. In this sense, a meta-analysis supports that frozen section histopathology performed well in predicting a diagnosis of culture-positive periprosthetic PJI but had moderate accuracy in ruling out this diagnosis [8]. This finding is in agreement with those of our study, as we did not find differences between both tests.

According to the preoperative parameters, an interesting finding was that the predictive power of individual tests, such as the CRP and synovial fluid culture, which was higher for delayed infections than for other types of PJI. Although our study was related to different types of onset of PJI, the predictive power of CRP was not as good as reported by others [30]. Fink et al. [27] found synovial biopsy to have the highest diagnostic value for identification of late prosthetic knee infection compared to joint aspiration and CRP. However, a biopsy was performed only in patients with loose components who were more likely to be infected and at times, multiple biopsies were necessary [27, 31]. Therefore, a synovial biopsy, with sensitivities ranging from 65% to 100% [27–32], could be an option for late PJI in which synovial fluid was not diagnostic, but there was a strong suspicion of infection. However, ultrasound-guided joint aspiration, with sensitivities varying widely (12–100%) would be a reasonable approach in patients with delayed/late infections and stable implants [27].

Regarding the combination of techniques, our main finding was that the conjunction of histopathology and sonicate fluid culture yielded a sensitivity of 100% for all types of infection with specificities ranging from 95.8% to 100%. By combining histopathology and peri-implant tissue, all early, 97% of delayed and 94.8% of late cases were diagnosed with almost 100% specificity.

The combined measurement of preoperative parameters such as CRP and synovial fluid produced the highest preoperative predictive power for diagnosing late cases with a marked trend for delayed PJI. Recent studies have evaluated the measurement of synovial CRP in synovial fluid; however, the use of this measurement seems limited and offers no additional diagnostic information beyond serum CRP, which is already widely clinically available [33].

Results of the present study emphasize the idea that PJI is typically a biofilm-associated infection. In this regard, the Infectious Diseases Society of America (IDSA) suggests a gap in the validation of the diagnostic value of sonicate fluid culture and requires a higher level of evidence [3]. In the present study, peri-implant tissue culture was found to be the third best individual method, after sonicate fluid culture and histology, for the diagnosis of PJI.

The limitations of our study are first, it is a single-center study, with potential for uncontrolled selection biases, however, there is no variation in the methodologies, something that does not occur when drawing patients from multiple centers. Second, due to the small sample size for early and delayed PJI, the study may have lacked the ability to detect slight differences among these two types of infection and the diagnostic techniques compared and, some results approached the borderline of significance; also the small sample size may be a limitation towards conducting further analysis by the operative site (hip vs knee), with a larger sample size we might have been able to draw stronger conclusions. Third, the synovial fluid (SF) white cell count (WCC) and polymorphonuclear cell counts were not performed. Several studies have incorporated the SF analysis into a set of diagnostic criteria for PJI. However, it was found that SF-WCC had high statistical heterogeneity depending on the time at which the sample was obtained (preoperative vs intraoperative) and the operative site (hip vs knee). On the other hand, it may not be reliable for a patient with inflammatory arthropathy, the true diagnostic ability of this test depends on whether the synovial fluid aspiration is successful and the optimal cut-off values of these tests may need further large-scale validation. Fourth, a contributing problem is that CRP was acquired in 70% of overall PJI cases and preoperative aspiration of synovial fluid was performed in only 45% of overall PJI patients. Fifth, molecular microbiological methods were not performed, while the value of Polymerase Chain Reaction (PCR) in most studies is based on locally-developed PCR methods that can present unsatisfactory standardization which is why the development of a commercial system that features higher reproducibility would improve PJI diagnosis in most laboratories*.* However, multiplex PCR of sonication fluid, despite the promising results in PJI, is an expensive test and unfortunately is not commonly available in most clinical laboratories.

## Conclusions

This study demonstrates that for early PJI, sonication fluid and peri-implant tissue cultures are highly sensitive and reach the same accuracy. For delayed and late cases, sonication fluid is the most sensitive individual diagnostic method. The CRP, as a preoperative parameter, provided more accuracy in diagnosing delayed infections. The combination of a positive histology and sonicate fluid culture allows the diagnosis of all types of infection, and the combination of preoperative parameters, such as CRP and synovial fluid culture, significantly improved the diagnosis of late PJI and approached the borderline of significance for delayed PJI. Therefore, the observed results could have important implications for the accuracy in diagnosis of each type of PJI based on the onset of infection.

## Abbreviations

CFU: Colony-forming unit
CI: Confidence intervals
CNS: Coagulase-negative staphylococci
CRP: C reactive protein
ESBL: Extended-spectrum beta-lactamases
ESR: Erythrocyte sedimentation rate
GNB: Gram negative bacilli
IDSA: Infectious Diseases Society of America
NPV: Negative predictive value
PCR: Polymerase Chain Reaction
PJI: Prosthetic joint infection
PPV: Positive predictive value
ROC: Receiver-operating-characteristic
